# Crystal structure of di­chlorido-1κ*Cl*,2κ*Cl*-(μ_2_-3,5-dimethyl-1*H*-pyrazolato-1κ*N* ^2^:2κ*N* ^1^)(3,5-dimethyl-1*H*-pyrazole-2κ*N* ^2^){μ-2-[(2-hy­droxy­eth­yl)amino-1κ^2^ *N*,*O*]ethano­lato-1:2κ^2^ *O*:*O*}dicopper(II)

**DOI:** 10.1107/S2056989020011184

**Published:** 2020-08-25

**Authors:** Oleksandr S. Vynohradov, Vadim A. Pavlenko, Inna S. Safyanova, Kateryna Znovjyak, Sergiu Shova, Safarmamad M. Safarmamadov

**Affiliations:** a Department of Chemistry, Taras Shevchenko National University of Kyiv, Volodymyrska str. 64/13, 01601 Kyiv, Ukraine; b "Poni Petru" Institute of Macromolecular Chemistry, Aleea Gr. Ghica, Voda 41A, 700487 Iasi, Romania; c Department of Chemistry, Tajik National University, 17, Rudaki Avenue, Dushanbe, 734025, Tajikistan

**Keywords:** copper, copper complexes, crystal structure, pyrazole, di­ethano­lamine, X-ray crystallography, amino­alcohol ligand

## Abstract

The title compound, [Cu_2_(C_5_H_7_N_2_)(C_4_H_10_NO_2_)Cl_2_(C_5_H_8_N_2_)], is a pyrazolate amino­alcohol complex which contains two di­methyl­pyrazole mol­ecules in monodentate and bidentate-bridged coordination modes and a monodeproton­ated di­ethano­lamine mol­ecule. Both copper atoms are involved in the formation of non-planar five-membered chelate rings. One Cu atom is in a distorted tetra­hedral environment formed by the pyridine nitro­gen atom of the protonated di­methyl­pyrazole mol­ecule, the N atom of the deprotonated bridged di­methyl­pyrazole, the Cl atom and the bridged O atom of the monodeprotonated di­ethano­lamine. The second Cu atom has an inter­mediate environment between trigonal bipyramidal and square pyramidal, formed by the N atom of the deprotonated bridged di­methyl­pyrazole, the Cl atom and the N atom of the amino­alcohol, and two O atoms of the deprotonated and protonated OH groups. In the crystal, N—H⋯Cl hydrogen bonds link the molecules into anti­symmetric chains running along the *a*-axis direction. Adjacent chains are connected by O—H⋯O hydrogen bonds involving the hydroxyl group as donor.

## Abstract 2

The title pyrazolate amino­alcohol complex comprises two di­methyl­pyrazole mol­ecules in monodentate and bidentate-bridged coordination modes and a monodeprotonated di­ethano­lamine mol­ecule.

## Chemical context   

**Figure 1:**
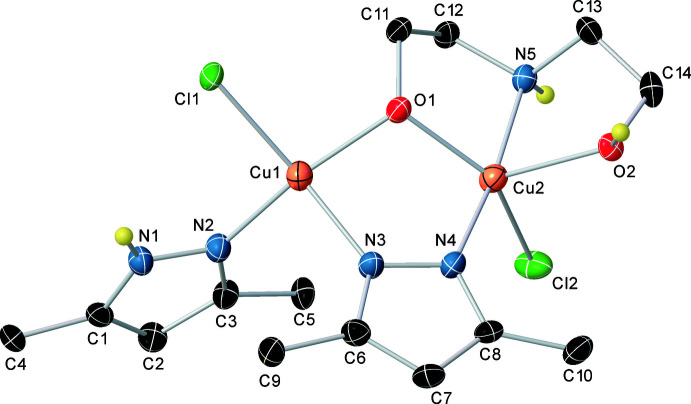
The mol­ecular structure of the title compound with displacement ellipsoids drawn at the 50% probability level.

Metal complexes of paramagnetic metal ions formed by polynucleative or polydentate ligands are of great inter­est as they often exhibit non-trivial magnetic behaviour (Gumienna-Kontecka *et al.*, 2007 ▸; Suleimanov *et al.*, 2015 ▸; Gural’skiy *et al.*, 2012 ▸). Among polydentate and polynucleative ligands, those containing both nitro­gen and oxygen donor atoms are probably the most versatile and efficient chelators for the vast majority of metal ions (Pavlishchuk *et al.*, 2010 ▸, 2011 ▸; Strotmeyer *et al.*, 2003 ▸). Amino alcohol ligands and their derivatives are one of the most widely used representatives of *N*,*O*-chelators and attract attention as strong polydentate ligands that can form coordination compounds with transition metals (Hughes *et al.*, 1972 ▸). Amino alcohols contain both amino and hydroxyl groups within the same mol­ecule, and therefore they are good chelating and bridging ligands. Polynuclear complexes of 3*d* metals with amino alcohols or their deprotonated forms can show non-trivial properties as catalysts, materials with different magnetic properties or biologically active compounds (Reiter *et al.*, 2006 ▸). Amino alcohol ligands are used to prepare copper(II) amino alcoholates that can self-assemble to form both mono- and multinuclear complexes. In bionuclear copper complexes, the metal atoms can be connected by bridged oxygen atoms (alk­oxy) from two different di­ethano­lamine mol­ecules (Tudor *et al.*, 2003 ▸; Marin *et al.*, 2005 ▸), or combined by a single oxygen atom from an amino alcohol and a bridged ligand mol­ecule (Ashurov *et al.*, 2015 ▸). There are several typical binding modes of tridentate amino alcohol ligands to copper(II) ions and other metals such as lanthanides, yttrium, and alkaline-earth metals (Breeze & Wang, 1994 ▸; Chen *et al.*, 1995 ▸; Wang *et al.*, 1995 ▸). It is a well-known fact that copper coordination compounds can be modified with amino alcohols. For example, copper complexes with theophylline show promising potential anti­tumor action and can be modified with di­ethano­lamine by similar coord­in­ation of amino alcohols to the copper atom (Madarász *et al.*, 2000 ▸). Studies of both tridentate- and bidentate-coordinated amino alcohol ligands to the copper atom have been carried out (Wang, 1995 ▸). Complexes of 3*d* metals with a tricoordinated di­ethano­lamine are inter­esting objects for synthesis and further studies (Buvaylo *et al.*, 2009 ▸). Considering the above, we understand the importance of accumulating a theoretical information base on such coordination compounds, and therefore in this article we report the synthesis and crystal structure of a new binuclear mixed-ligand copper(II) complex containing 3,5-di­methyl­pyrazole and di­ethano­lamine (Fig. 1 ▸).
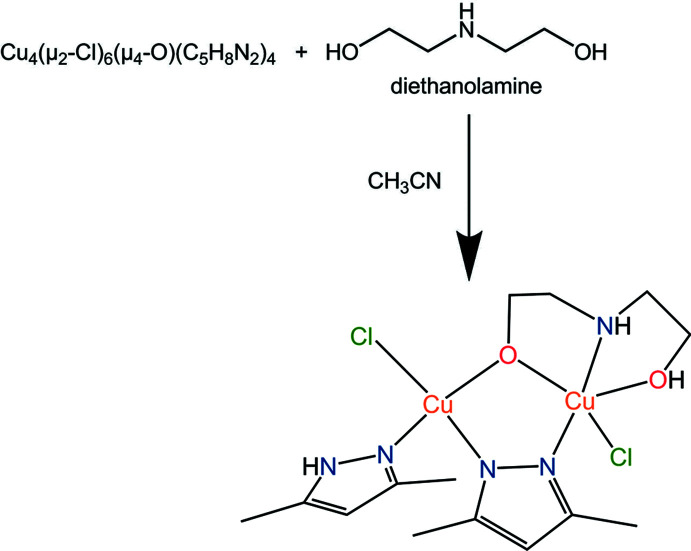


## Structural commentary   

**Figure 2:**
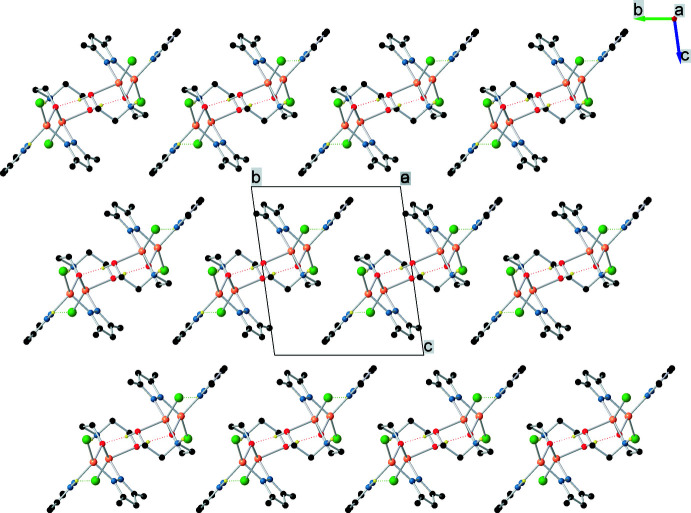
The crystal packing of the title compound viewed along the *a-*axis direction.

The crystal structure of the title compound (Fig. 2 ▸) consists of dinuclear Cu_2_(Hdmpz)(dmpz-H)(HDEA)Cl_2_ (Hdmpz = 3,5-dimethyl-1*H*-pyrazole, dmpz-H = deprotonated 3,5-dimethyl-pyrazole, and HDEA = monodeprotonated di­ethano­lamine) units enclosed in two anti­symmetrically oriented rows along the *a* axis. The unit cell consists of two unrelated structural fragments from both rows (Fig. 2 ▸). Along the *a* axis within one row, each mol­ecule is bonded to the preceding and subsequent ones by hydrogen bonds of the same length. Along the *b* axis, the formation of mol­ecules into dimers is due to hydrogen bonds of equal length between the bridged oxygen atom and the non-deprotonated hy­droxy group of the adjacent mol­ecule. The title dinuclear pyrazolate amino-alcohol compound forms a cyclic structure. Two copper atoms bridged by an oxygen atom of a deprotonated di­ethano­lamine and by a mol­ecule of deprotonated di­methyl­pyrazole form a five-membered bimetallic ring. The five-membered metallocycle has a non-planar structure. The N atoms of the bridging mol­ecule of di­methyl­pyrazole are in the plane of the metallocycle while the bridging O atom is out of this plane by 0.802 (1) Å. The angle between the Cu1/O1/Cu2 and Cu1/Cu2/N3/N4 planes is 45.85 (8)°. The geometrical environment of Cu1 with a coordination number of 4 is different from that of Cu2, which exhibits a coordination number of 5. The Cu1 atom is in a distorted tetra­hedral environment formed by the pyridine N atom of the non-deprotonated di­methyl­pyrazole mol­ecule, the N atom of the deprotonated bridging di­methyl­pyrazole, the Cl atom and the bridging O atom of the monodeprotonated di­ethano­lamine. The environment of the Cu2 metal center is inter­mediate between trigonal bipyramidal and square pyramidal, formed by the N atom of the deprotonated bridged di­methyl­pyrazole, the Cl atom and the amino­alcohol N atom, and two O atoms of the deprotonated and non-deprotonated OH groups. The inter­metallic distance between Cu1 and Cu2 is 3.2439 (4) Å. The di­ethano­lamine fragment is coordinated by all donor atoms to copper in a tri­dentate mode (with atom O1 bridging the two metal centers Cu1 and Cu2) and forms two similar non-planar five-membered metallocycles. It is worth mentioning that the Cu1—O1 distance of 1.9388 (13) Å (Table 1 ▸) differs significantly from the Cu2—O2 distance of 2.2441 (14) Å.

## Supra­molecular features   

**Figure 3:**
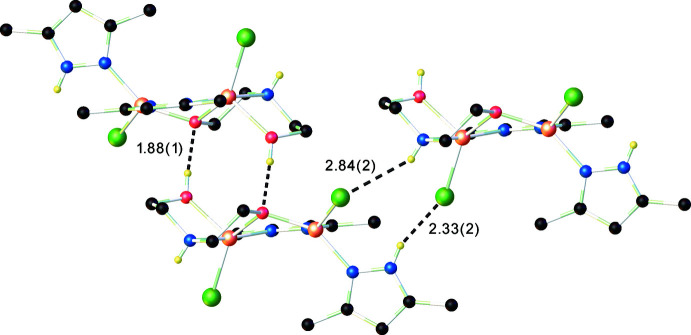
The hydrogen bonds (dotted lines) in the crystal structure of the title compound.

**Figure 4:**
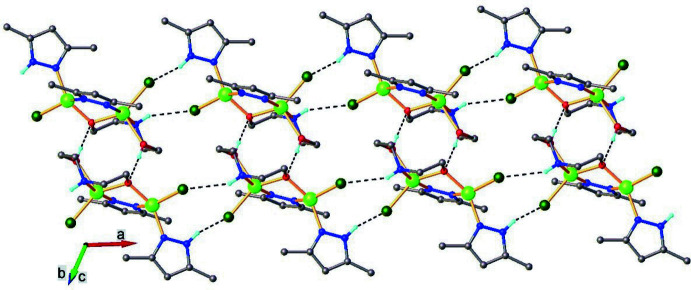
Partial view of the one-dimensional architecture in the crystal structure of the title compound. Non-relevant H atoms are omitted for clarity.

In the crystal, hydrogen bonds (Table 2 ▸) are observed between the N and Cl atoms (N1—H1⋯Cl2 and N5–H5⋯Cl1) leading to the formation of anti­symmetric chains running along the *a*-axis direction (Fig. 3 ▸). Adjacent chains are connected by hydrogen bonds between the hydroxyl group as donor and the O1 atom of the adjacent mol­ecule as acceptor. There are different fragments that are potential H-atom donors or acceptors and further analysis of the structure indicates the presence of multiple non-covalent inter­molecular inter­actions. The crystal structure (Fig. 4 ▸) consists of discrete parallel-packed one-dimensional supramolecular formations, which are assembled by connecting two infinite chains (formed by N—H⋯Cl hydrogen bonds) *via* O—H⋯O hydrogen-bonding inter­actions.

**Figure 5:**
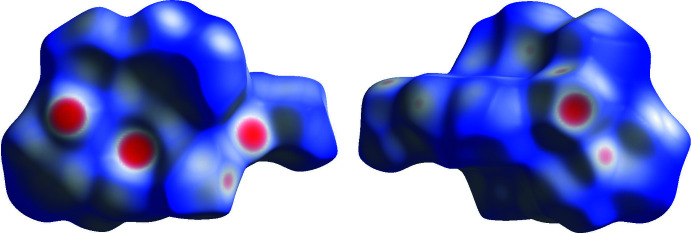
Two projections of Hirshfeld surfaces mapped over *d*_norm_ showing the inter­molecular inter­actions within the mol­ecule. Red areas represent contacts shorter than the sum of the van der Waals radii, while blue areas represent regions where contacts are longer than the sum of van der Waals radii, and white areas are zones close to the sum of van der Waals radii.

**Figure 6:**
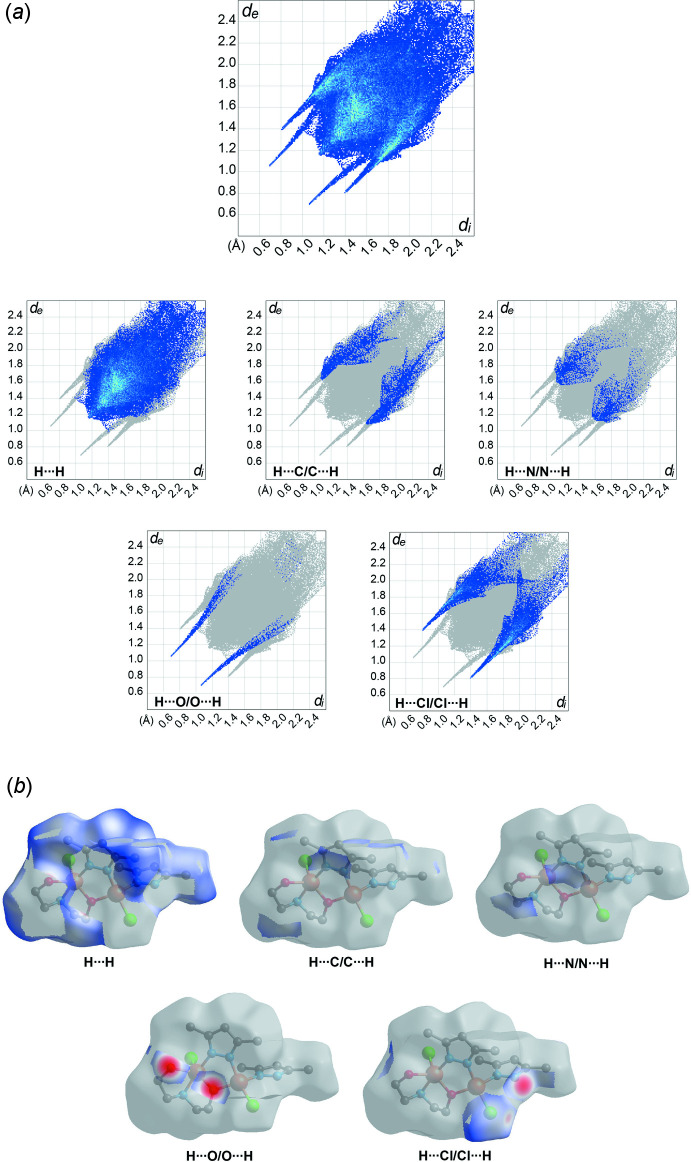
(*a*) The overall two-dimensional fingerprint plot and those delineated into specified inter­actions. (*b*) Hirshfeld surface representations with the function *d*_norm_ plotted onto the surface for the different inter­actions.

The Hirshfeld surface analysis and the associated two-dimensional fingerprint plots were generated using *Crystal Explorer 17.5* software (Turner *et al.*, 2018 ▸), with a standard resolution of the three-dimensional *d*_norm_ surfaces plotted over a fixed colour scale of −0.6711 (red) to 1.7846 (blue) a.u. The pale-red spots in Fig. 5 ▸ represent short contacts and negative *d*_norm_ values on the surface corresponding to the inter­actions described above. The overall two-dimensional fingerprint plot is illustrated in Fig. 6 ▸ *a*. The Hirshfeld surfaces mapped over *d*_norm_ are shown for the H⋯H, H⋯C/C⋯H, H⋯Cl/Cl⋯H, H⋯O/O⋯H, and H⋯N/N⋯H contacts, and the two-dimensional fingerprint plots are given in Fig. 6 ▸ *b*. At 64.1%, the largest contribution to the overall crystal packing comes from H⋯H inter­actions, which are located in the middle region of the fingerprint plot. H⋯C/C⋯H contacts contribute 8.2%, and H⋯N/N⋯H contacts contribute 2.4% to the Hirshfeld surface, both resulting in a pair of characteristic wings. The H⋯O/O⋯H contacts make a 2.7% contribution, forming the inner sharp tips of the Hirshfeld surface, while H⋯Cl/Cl⋯H contacts contribute 19.1% and form the outer sharp tips in the fingerprint plot.

## Database survey   

A search of the Cambridge Structural database (CSD version 5.41; November 2019; Groom *et al.*, 2016 ▸) for the CuNH(CCO)_2_ moiety (di­ethano­lamine is coordinated to the copper atom) gave 168 hits. Most similar to the title compound are the dinuclear complexes with coordinated di­ethano­lamine mol­ecules, and copper atoms connected by a bridging oxygen atom and some other ligands, see: refcodes ELESAP (Tudor *et al.*, 2003 ▸), FARKAL (Marin *et al.*, 2005 ▸) and WITBAC (Madarász *et al.*, 2000 ▸).

## Synthesis and crystallization   

A 1.76 mmol di­ethano­lamine solution was added dropwise to a 1.15 mmol aceto­nitrile solution of complex Cu_4_(μ_2_-Cl)_6_(μ_4_-O)(C_5_H_8_N_2_)_4_ under stirring. The mixture was stirred for a further 2 h with oxygen access and without heating. Amino alcohol was added to the brown solution and the colour of the mixture changed to green. Dark-green crystals of the title compound suitable for a single crystal X-ray analysis were obtained in 55% yield by slow gas diffusion in an aceto­nitrile/hexane isolated system. Elemental analysis of C_14_H_25_Cl_2_Cu_2_N_5_O_2_: found C 33.96, H 5.267 and N 14.13% (calculated C 34.08, H 5.1, N 14.19%). The starting compound Cu_4_(μ_2_-Cl)_6_(μ_4_-O)(C_5_H_8_N_2_)_4_ is a polymorphic modification of the already known tetra­nuclear copper pyrazole-containing cluster Cu_4_OCl_6_(C_5_H_8_N_2_)_4_ and was obtained from the Cu–CuCl_2_·2H_2_O–Hdmpz system.

## Refinement   

Crystal data, data collection and structure refinement details are summarized in Table 3 ▸. C-bound H atoms were placed in calculated positions (C—H = 0.93–0.97 Å) and refined using a riding model with *U*_iso_(H) = 1.2*U*_eq_(C) or 1.5*U*_eq_(C-meth­yl). The O2—H2*A* distance was restrained to 0.85±0.01 Å. The N and O atoms were refined with *U*_iso_(H) = 1.2*U*_eq_(N) or 1.5*U*_eq_(O).

## Supplementary Material

Crystal structure: contains datablock(s) I. DOI: 10.1107/S2056989020011184/zq2256sup1.cif

Structure factors: contains datablock(s) I. DOI: 10.1107/S2056989020011184/zq2256Isup2.hkl

Supporting information file. DOI: 10.1107/S2056989020011184/zq2256Isup5.mol

here is checkcif pdf file. DOI: 10.1107/S2056989020011184/zq2256sup3.pdf

here is the IR-spectrum of title compound. DOI: 10.1107/S2056989020011184/zq2256sup4.txt

here is the pdf version of the article. DOI: 10.1107/S2056989020011184/zq2256sup6.pdf

CCDC reference: 2023401

Additional supporting information:  crystallographic information; 3D view; checkCIF report

## Figures and Tables

**Table 1:** Selected bond lengths (Å)

Cu1—Cl1	2.2403 (6)	Cu2—N4	1.9268 (17)
Cu1—N2	1.9635 (16)	Cu2—N5	1.9916 (17)
Cu1—N3	1.9770 (17)	Cu2—O1	2.0001 (13)
Cu1—O1	1.9388 (13)	Cu2—O2	2.2441 (14)
Cu2—Cl2	2.2937 (6)		

**Table 2:** Hydrogen-bond geometry (Å, °)

*D*—H⋯*A*	*D*—H	H⋯*A*	*D*⋯*A*	*D*—H⋯*A*
N1—H1⋯Cl2^i^	0.87 (2)	2.33 (2)	3.1201 (18)	152 (2)
N5—H5⋯Cl1^ii^	0.80 (2)	2.84 (2)	3.5593 (18)	150 (2)
O2—H2*A*⋯O1^iii^	0.85 (1)	1.88 (1)	2.7264 (19)	174 (2)

Symmetry codes: (i) 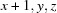; (ii) 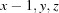; (iii) 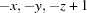.

**Table 3:** Experimental details

Crystal data	
Chemical formula	[Cu_2_(C_5_H_7_N_2_)(C_4_H_10_NO_2_)Cl_2_(C_5_H_8_N_2_)]
*M*_r_	493.37
Crystal system, space group	Triclinic, *P* 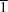
Temperature (K)	180
*a*, *b*, *c* (Å)	9.0732 (5), 10.7460 (6), 11.5578 (6)
α, β, γ (°)	92.373 (4), 102.383 (5), 112.703 (5)
*V* (Å^3^)	1005.70 (10)
*Z*	2
Radiation type	Mo *K*α
μ (mm^−1^)	2.40
Crystal size (mm)	0.4 × 0.3 × 0.3

Data collection	
Diffractometer	Rigaku Oxford Diffraction Xcalibur, Eos
Absorption correction	Multi-scan (*CrysAlis PRO*; Rigaku OD, 2019 ▸)
*T*_min_, *T*_max_	0.553, 1.000
No. of measured, independent and observed [*I* > 2σ(*I*)] reflections	8833, 4681, 4108
*R*_int_	0.018
(sin θ/λ)_max_ (Å^−1^)	0.693

Refinement	
*R*[*F* ^2^ > 2σ(*F* ^2^)], *wR*(*F* ^2^), *S*	0.027, 0.061, 1.05
No. of reflections	4681
No. of parameters	239
No. of restraints	3
H-atom treatment	H atoms treated by a mixture of independent and constrained refinement
Δρ_max_, Δρ_min_ (e Å^−3^)	0.36, −0.43

Computer programs: *CrysAlis PRO* (Rigaku OD, 2019 ▸), *SHELXT* (Sheldrick, 2015*a* ▸), *SHELXL2018/3* (Sheldrick, 2015*b* ▸) and *OLEX2* (Dolomanov *et al.*, 2009 ▸).

## supplementary crystallographic information

### Crystal data

**Table:**

[Cu_2_(C_5_H_7_N_2_)(C_4_H_10_NO_2_)Cl_2_(C_5_H_8_N_2_)]	*Z* = 2
*M**_r_* = 493.37	*F*(000) = 504
Triclinic, *P*1	*D*_x_ = 1.629 Mg m^−^^3^
*a* = 9.0732 (5) Å	Mo *K*α radiation, λ = 0.71073 Å
*b* = 10.7460 (6) Å	Cell parameters from 4259 reflections
*c* = 11.5578 (6) Å	θ = 1.8–29.2°
α = 92.373 (4)°	µ = 2.40 mm^−^^1^
β = 102.383 (5)°	*T* = 180 K
γ = 112.703 (5)°	Prism, clear intense green
*V* = 1005.70 (10) Å^3^	0.4 × 0.3 × 0.3 mm

### Data collection

**Table:**

Rigaku Oxford Diffraction Xcalibur, Eos diffractometer	4681 independent reflections
Radiation source: fine-focus sealed X-ray tube, Enhance (Mo) X-ray Source	4108 reflections with *I* > 2σ(*I*)
Graphite monochromator	*R*_int_ = 0.018
Detector resolution: 16.1593 pixels mm^-1^	θ_max_ = 29.5°, θ_min_ = 1.8°
ω scans	*h* = −11→10
Absorption correction: multi-scan (CrysAlisPro; Rigaku OD, 2019)	*k* = −13→13
*T*_min_ = 0.553, *T*_max_ = 1.000	*l* = −15→15
8833 measured reflections	

### Refinement

**Table:**

Refinement on *F*^2^	Primary atom site location: dual
Least-squares matrix: full	Hydrogen site location: mixed
*R*[*F*^2^ > 2σ(*F*^2^)] = 0.027	H atoms treated by a mixture of independent and constrained refinement
*wR*(*F*^2^) = 0.061	*w* = 1/[σ^2^(*F*_o_^2^) + (0.0223*P*)^2^ + 0.5005*P*] where *P* = (*F*_o_^2^ + 2*F*_c_^2^)/3
*S* = 1.05	(Δ/σ)_max_ = 0.001
4681 reflections	Δρ_max_ = 0.36 e Å^−^^3^
239 parameters	Δρ_min_ = −0.43 e Å^−^^3^
3 restraints	

### Special details

**Table:**

Geometry. All esds (except the esd in the dihedral angle between two l.s. planes) are estimated using the full covariance matrix. The cell esds are taken into account individually in the estimation of esds in distances, angles and torsion angles; correlations between esds in cell parameters are only used when they are defined by crystal symmetry. An approximate (isotropic) treatment of cell esds is used for estimating esds involving l.s. planes.

### Fractional atomic coordinates and isotropic or equivalent isotropic displacement parameters (Å^2^)

**Table:**

	*x*	*y*	*z*	*U*_iso_*/*U*_eq_	
C1	0.8325 (3)	0.5970 (2)	0.87558 (18)	0.0213 (4)	
C2	0.7173 (3)	0.6360 (2)	0.9068 (2)	0.0303 (5)	
H2	0.737259	0.705862	0.966030	0.036*	
C3	0.5648 (3)	0.5504 (2)	0.8325 (2)	0.0249 (5)	
C4	1.0153 (3)	0.6516 (2)	0.9206 (2)	0.0299 (5)	
H4A	1.053223	0.583541	0.900423	0.045*	
H4B	1.045118	0.675213	1.005925	0.045*	
H4C	1.065349	0.731079	0.884536	0.045*	
C5	0.3987 (3)	0.5503 (3)	0.8250 (3)	0.0427 (7)	
H5A	0.360893	0.577420	0.750146	0.064*	
H5B	0.405616	0.612959	0.889610	0.064*	
H5C	0.322664	0.460389	0.830295	0.064*	
C6	0.3858 (3)	0.1471 (2)	0.84323 (18)	0.0222 (5)	
C7	0.2607 (3)	0.0678 (2)	0.89308 (19)	0.0237 (5)	
H7	0.271158	0.021651	0.958574	0.028*	
C8	0.1163 (3)	0.0714 (2)	0.82511 (17)	0.0189 (4)	
C9	0.5663 (3)	0.1797 (3)	0.8802 (2)	0.0411 (7)	
H9A	0.604540	0.168147	0.810855	0.062*	
H9B	0.585441	0.119540	0.934917	0.062*	
H9C	0.624799	0.272188	0.918419	0.062*	
C10	−0.0560 (3)	0.0091 (2)	0.8396 (2)	0.0265 (5)	
H10A	−0.073035	0.071365	0.891799	0.040*	
H10B	−0.072923	−0.073948	0.873356	0.040*	
H10C	−0.132710	−0.010057	0.762932	0.040*	
C11	0.1643 (3)	0.2883 (2)	0.41712 (17)	0.0192 (4)	
H11A	0.113125	0.213856	0.351586	0.023*	
H11B	0.265007	0.353315	0.401922	0.023*	
C12	0.0478 (3)	0.3565 (2)	0.42571 (19)	0.0209 (4)	
H12A	0.107248	0.443231	0.477042	0.025*	
H12B	0.002000	0.373194	0.347020	0.025*	
C13	−0.2144 (2)	0.1487 (2)	0.39115 (18)	0.0210 (4)	
H13A	−0.163870	0.108472	0.343581	0.025*	
H13B	−0.289446	0.177492	0.337517	0.025*	
C14	−0.3067 (3)	0.0466 (2)	0.4624 (2)	0.0234 (5)	
H14A	−0.363395	0.085052	0.505581	0.028*	
H14B	−0.388495	−0.033863	0.409321	0.028*	
Cl1	0.57083 (6)	0.33924 (5)	0.50253 (4)	0.02235 (12)	
Cl2	−0.05593 (7)	0.31818 (6)	0.74647 (5)	0.02968 (14)	
Cu1	0.41923 (3)	0.31469 (2)	0.63633 (2)	0.01473 (7)	
Cu2	0.02665 (3)	0.20789 (2)	0.61602 (2)	0.01395 (7)	
N1	0.7493 (2)	0.49360 (18)	0.78604 (15)	0.0175 (4)	
H1	0.788 (3)	0.446 (2)	0.749 (2)	0.021*	
N2	0.5853 (2)	0.46264 (17)	0.75898 (15)	0.0182 (4)	
N3	0.3203 (2)	0.19520 (17)	0.74924 (14)	0.0172 (4)	
N4	0.1545 (2)	0.14748 (17)	0.73807 (14)	0.0166 (3)	
N5	−0.0860 (2)	0.26688 (17)	0.47523 (15)	0.0156 (3)	
H5	−0.133 (3)	0.308 (2)	0.499 (2)	0.019*	
O1	0.20146 (16)	0.23756 (13)	0.52738 (11)	0.0144 (3)	
O2	−0.19285 (18)	0.01036 (14)	0.54515 (13)	0.0228 (3)	
H2A	−0.200 (2)	−0.0673 (12)	0.5176 (19)	0.034*	

### Atomic displacement parameters (Å^2^)

**Table:**

	*U*^11^	*U*^22^	*U*^33^	*U*^12^	*U*^13^	*U*^23^
C1	0.0227 (11)	0.0187 (11)	0.0162 (10)	0.0030 (8)	0.0029 (8)	0.0002 (8)
C2	0.0311 (13)	0.0258 (12)	0.0283 (12)	0.0063 (10)	0.0088 (10)	−0.0104 (10)
C3	0.0250 (12)	0.0215 (11)	0.0293 (12)	0.0088 (9)	0.0111 (9)	−0.0017 (9)
C4	0.0221 (12)	0.0329 (13)	0.0233 (12)	0.0027 (10)	−0.0004 (9)	−0.0024 (10)
C5	0.0312 (15)	0.0431 (16)	0.0576 (18)	0.0179 (12)	0.0162 (13)	−0.0080 (14)
C6	0.0232 (11)	0.0251 (12)	0.0208 (11)	0.0128 (9)	0.0044 (8)	0.0053 (9)
C7	0.0303 (12)	0.0264 (12)	0.0174 (10)	0.0135 (10)	0.0071 (9)	0.0093 (9)
C8	0.0247 (11)	0.0188 (10)	0.0149 (10)	0.0090 (9)	0.0076 (8)	0.0027 (8)
C9	0.0271 (14)	0.0578 (19)	0.0427 (15)	0.0214 (13)	0.0059 (11)	0.0249 (13)
C10	0.0276 (12)	0.0299 (12)	0.0251 (11)	0.0108 (10)	0.0132 (9)	0.0106 (9)
C11	0.0163 (10)	0.0270 (11)	0.0155 (10)	0.0085 (9)	0.0058 (8)	0.0080 (8)
C12	0.0192 (11)	0.0214 (11)	0.0231 (11)	0.0078 (8)	0.0067 (8)	0.0115 (9)
C13	0.0162 (10)	0.0233 (11)	0.0206 (10)	0.0076 (8)	0.0003 (8)	−0.0015 (9)
C14	0.0148 (10)	0.0187 (11)	0.0344 (12)	0.0053 (8)	0.0054 (9)	−0.0027 (9)
Cl1	0.0171 (3)	0.0277 (3)	0.0228 (3)	0.0076 (2)	0.00912 (19)	0.0017 (2)
Cl2	0.0361 (3)	0.0433 (3)	0.0217 (3)	0.0284 (3)	0.0089 (2)	−0.0001 (2)
Cu1	0.01202 (13)	0.01529 (13)	0.01474 (12)	0.00332 (9)	0.00345 (9)	0.00108 (9)
Cu2	0.01386 (13)	0.01565 (13)	0.01402 (12)	0.00676 (10)	0.00508 (9)	0.00341 (9)
N1	0.0144 (9)	0.0178 (9)	0.0193 (9)	0.0061 (7)	0.0034 (7)	−0.0003 (7)
N2	0.0153 (9)	0.0193 (9)	0.0192 (9)	0.0059 (7)	0.0052 (7)	0.0006 (7)
N3	0.0149 (9)	0.0198 (9)	0.0177 (8)	0.0073 (7)	0.0047 (7)	0.0048 (7)
N4	0.0160 (9)	0.0173 (9)	0.0175 (8)	0.0063 (7)	0.0069 (7)	0.0041 (7)
N5	0.0144 (9)	0.0148 (9)	0.0192 (9)	0.0068 (7)	0.0060 (7)	0.0014 (7)
O1	0.0129 (7)	0.0162 (7)	0.0143 (7)	0.0053 (5)	0.0046 (5)	0.0047 (5)
O2	0.0245 (8)	0.0128 (7)	0.0268 (8)	0.0043 (6)	0.0040 (6)	0.0010 (6)

### Geometric parameters (Å, º)

**Table:**

C1—C2	1.372 (3)	C11—H11B	0.9700
C1—C4	1.490 (3)	C11—C12	1.517 (3)
C1—N1	1.341 (3)	C11—O1	1.432 (2)
C2—H2	0.9300	C12—H12A	0.9700
C2—C3	1.393 (3)	C12—H12B	0.9700
C3—C5	1.490 (3)	C12—N5	1.473 (3)
C3—N2	1.335 (3)	C13—H13A	0.9700
C4—H4A	0.9600	C13—H13B	0.9700
C4—H4B	0.9600	C13—C14	1.499 (3)
C4—H4C	0.9600	C13—N5	1.478 (2)
C5—H5A	0.9600	C14—H14A	0.9700
C5—H5B	0.9600	C14—H14B	0.9700
C5—H5C	0.9600	C14—O2	1.432 (3)
C6—C7	1.386 (3)	Cu1—Cl1	2.2403 (6)
C6—C9	1.494 (3)	Cu1—N2	1.9635 (16)
C6—N3	1.344 (3)	Cu1—N3	1.9770 (17)
C7—H7	0.9300	Cu1—O1	1.9388 (13)
C7—C8	1.391 (3)	Cu2—Cl2	2.2937 (6)
C8—C10	1.494 (3)	Cu2—N4	1.9268 (17)
C8—N4	1.341 (3)	Cu2—N5	1.9916 (17)
C9—H9A	0.9600	Cu2—O1	2.0001 (13)
C9—H9B	0.9600	Cu2—O2	2.2441 (14)
C9—H9C	0.9600	N1—H1	0.87 (2)
C10—H10A	0.9600	N1—N2	1.353 (2)
C10—H10B	0.9600	N3—N4	1.363 (2)
C10—H10C	0.9600	N5—H5	0.80 (2)
C11—H11A	0.9700	O2—H2A	0.853 (9)
			
C2—C1—C4	131.7 (2)	N5—C12—H12B	109.9
N1—C1—C2	106.26 (19)	H13A—C13—H13B	108.4
N1—C1—C4	122.0 (2)	C14—C13—H13A	110.0
C1—C2—H2	126.7	C14—C13—H13B	110.0
C1—C2—C3	106.60 (19)	N5—C13—H13A	110.0
C3—C2—H2	126.7	N5—C13—H13B	110.0
C2—C3—C5	129.4 (2)	N5—C13—C14	108.28 (16)
N2—C3—C2	109.4 (2)	C13—C14—H14A	109.8
N2—C3—C5	121.1 (2)	C13—C14—H14B	109.8
C1—C4—H4A	109.5	H14A—C14—H14B	108.3
C1—C4—H4B	109.5	O2—C14—C13	109.25 (16)
C1—C4—H4C	109.5	O2—C14—H14A	109.8
H4A—C4—H4B	109.5	O2—C14—H14B	109.8
H4A—C4—H4C	109.5	N2—Cu1—Cl1	96.92 (5)
H4B—C4—H4C	109.5	N2—Cu1—N3	96.00 (7)
C3—C5—H5A	109.5	N3—Cu1—Cl1	144.30 (5)
C3—C5—H5B	109.5	O1—Cu1—Cl1	98.89 (4)
C3—C5—H5C	109.5	O1—Cu1—N2	148.51 (7)
H5A—C5—H5B	109.5	O1—Cu1—N3	86.76 (6)
H5A—C5—H5C	109.5	N4—Cu2—Cl2	95.29 (5)
H5B—C5—H5C	109.5	N4—Cu2—N5	171.92 (7)
C7—C6—C9	129.3 (2)	N4—Cu2—O1	87.45 (6)
N3—C6—C7	108.86 (19)	N4—Cu2—O2	99.70 (6)
N3—C6—C9	121.9 (2)	N5—Cu2—Cl2	92.21 (5)
C6—C7—H7	127.0	N5—Cu2—O1	84.77 (6)
C6—C7—C8	105.95 (19)	N5—Cu2—O2	81.29 (6)
C8—C7—H7	127.0	O1—Cu2—Cl2	142.41 (4)
C7—C8—C10	130.0 (2)	O1—Cu2—O2	112.12 (5)
N4—C8—C7	108.08 (19)	O2—Cu2—Cl2	104.35 (4)
N4—C8—C10	121.88 (19)	C1—N1—H1	127.8 (15)
C6—C9—H9A	109.5	C1—N1—N2	111.74 (17)
C6—C9—H9B	109.5	N2—N1—H1	120.4 (15)
C6—C9—H9C	109.5	C3—N2—Cu1	129.29 (15)
H9A—C9—H9B	109.5	C3—N2—N1	105.97 (16)
H9A—C9—H9C	109.5	N1—N2—Cu1	124.74 (13)
H9B—C9—H9C	109.5	C6—N3—Cu1	132.39 (15)
C8—C10—H10A	109.5	C6—N3—N4	107.94 (17)
C8—C10—H10B	109.5	N4—N3—Cu1	119.66 (13)
C8—C10—H10C	109.5	C8—N4—Cu2	132.78 (14)
H10A—C10—H10B	109.5	C8—N4—N3	109.16 (16)
H10A—C10—H10C	109.5	N3—N4—Cu2	117.88 (13)
H10B—C10—H10C	109.5	C12—N5—C13	115.41 (16)
H11A—C11—H11B	108.3	C12—N5—Cu2	105.06 (12)
C12—C11—H11A	109.9	C12—N5—H5	111.3 (17)
C12—C11—H11B	109.9	C13—N5—Cu2	111.35 (13)
O1—C11—H11A	109.9	C13—N5—H5	106.2 (16)
O1—C11—H11B	109.9	Cu2—N5—H5	107.3 (16)
O1—C11—C12	108.86 (16)	C11—O1—Cu1	122.25 (12)
C11—C12—H12A	109.9	C11—O1—Cu2	111.54 (11)
C11—C12—H12B	109.9	Cu1—O1—Cu2	110.88 (6)
H12A—C12—H12B	108.3	C14—O2—Cu2	104.53 (12)
N5—C12—C11	108.97 (17)	C14—O2—H2A	108.4 (13)
N5—C12—H12A	109.9	Cu2—O2—H2A	131.5 (13)
			
C1—C2—C3—C5	−177.8 (3)	C9—C6—C7—C8	179.7 (2)
C1—C2—C3—N2	0.1 (3)	C9—C6—N3—Cu1	−0.9 (3)
C1—N1—N2—C3	0.9 (2)	C9—C6—N3—N4	179.7 (2)
C1—N1—N2—Cu1	−178.30 (15)	C10—C8—N4—Cu2	2.8 (3)
C2—C1—N1—N2	−0.8 (3)	C10—C8—N4—N3	177.58 (18)
C2—C3—N2—Cu1	178.58 (16)	C11—C12—N5—C13	−76.2 (2)
C2—C3—N2—N1	−0.5 (3)	C11—C12—N5—Cu2	46.87 (17)
C4—C1—C2—C3	178.4 (2)	C12—C11—O1—Cu1	−111.63 (16)
C4—C1—N1—N2	−179.0 (2)	C12—C11—O1—Cu2	22.96 (19)
C5—C3—N2—Cu1	−3.4 (3)	C13—C14—O2—Cu2	41.26 (17)
C5—C3—N2—N1	177.5 (2)	C14—C13—N5—C12	161.80 (17)
C6—C7—C8—C10	−177.5 (2)	C14—C13—N5—Cu2	42.16 (19)
C6—C7—C8—N4	1.0 (2)	Cu1—N3—N4—C8	−178.77 (13)
C6—N3—N4—C8	0.7 (2)	Cu1—N3—N4—Cu2	−3.11 (18)
C6—N3—N4—Cu2	176.41 (13)	N1—C1—C2—C3	0.5 (3)
C7—C6—N3—Cu1	179.33 (14)	N3—C6—C7—C8	−0.6 (2)
C7—C6—N3—N4	−0.1 (2)	N5—C13—C14—O2	−56.9 (2)
C7—C8—N4—Cu2	−175.87 (14)	O1—C11—C12—N5	−46.9 (2)
C7—C8—N4—N3	−1.1 (2)		

### Hydrogen-bond geometry (Å, º)

**Table:**

*D*—H···*A*	*D*—H	H···*A*	*D*···*A*	*D*—H···*A*
N1—H1···Cl2^i^	0.87 (2)	2.33 (2)	3.1201 (18)	152 (2)
N5—H5···Cl1^ii^	0.80 (2)	2.84 (2)	3.5593 (18)	150 (2)
O2—H2*A*···O1^iii^	0.85 (1)	1.88 (1)	2.7264 (19)	174 (2)

Symmetry codes: (i) *x*+1, *y*, *z*; (ii) *x*−1, *y*, *z*; (iii) −*x*, −*y*, −*z*+1.
