# Supplementary material for: Crystal structure of di­chlorido-1κCl,2κCl-(μ2-3,5-dimethyl-1H-pyrazolato-1κN 2:2κN 1)(3,5-dimethyl-1H-pyrazole-2κN 2){μ-2-[(2-hy­droxy­eth­yl)amino-1κ2 N,O]ethano­lato-1:2κ2 O:O}dicopper(II)
Source: Acta Crystallogr E Crystallogr Commun. 2020 Aug 25;76(Pt 9):1503–7. doi: 10.1107/S2056989020011184 (PMC7472753; doi:10.1107/S2056989020011184)

checkCIF/PLATON report

Structure factors have been supplied for datablock(s) shi_4306_

THIS REPORT IS FOR GUIDANCE ONLY. IF USED AS PART OF A REVIEW PROCEDURE FOR PUBLICATION, IT SHOULD NOT REPLACE THE EXPERTISE OF AN EXPERIENCED CRYSTALLOGRAPHIC REFEREE.

No syntax errors found.   CIF dictionary   Interpreting this report

Datablock: shi_4306_

```
Bond precision:  C-C = 0.0037 A          Wavelength=0.71073

Cell:           a=9.0732(5)       b=10.7460(6)      c=11.5578(6)
                alpha=92.373(4)   beta=102.383(5)   gamma=112.703(5)
Temperature:    180 K

                Calculated                Reported
Volume          1005.71(11)               1005.70(10)
Space group     P -1                      P -1
Hall group      -P 1                      -P 1
Moiety formula  C14 H25 Cl2 Cu2 N5 O2     C14 H25 Cl2 Cu2 N5 O2
Sum formula     C14 H25 Cl2 Cu2 N5 O2     C14 H25 Cl2 Cu2 N5 O2
Mr              493.39                    493.37
Dx,g cm-3       1.629                     1.629
Z               2                         2
Mu (mm-1)       2.397                     2.397
F000            504.0                     504.0
F000'           505.97
h,k,lmax        12,14,16                  11,13,15
Nref            5622                      4681
Tmin,Tmax       0.426,0.487               0.553,1.000
Tmin'           0.380

Correction method= # Reported T Limits: Tmin=0.553 Tmax=1.000
AbsCorr = MULTI-SCAN

Data completeness= 0.833          Theta(max)= 29.530

R(reflections)= 0.0266( 4108)     wR2(reflections)= 0.0613( 4681)

S = 1.054                  Npar= 239
```

The following ALERTS were generated. Each ALERT has the format
test-name_ALERT_alert-type_alert-level.
Click on the hyperlinks for more details of the test.

Alert level C

```
PLAT480_ALERT_4_C Long H...A H-Bond Reported H5       ..CL1      .       2.84 Ang.
```

Alert level G

```
PLAT002_ALERT_2_G Number of Distance or Angle Restraints on AtSite          4 Note
PLAT172_ALERT_4_G The CIF-Embedded .res File Contains DFIX Records          1 Report
PLAT173_ALERT_4_G The CIF-Embedded .res File Contains DANG Records          2 Report
PLAT232_ALERT_2_G Hirshfeld Test Diff (M-X)  Cu2      --N5       .        5.8 s.u.
PLAT793_ALERT_4_G Model has Chirality at N5          (Centro SPGR)          R Verify
PLAT794_ALERT_5_G Tentative Bond Valency for Cu2       (II)      .       2.23 Info
PLAT860_ALERT_3_G Number of Least-Squares Restraints .............          3 Note
PLAT912_ALERT_4_G Missing # of FCF Reflections Above STh/L=  0.600        810 Note
PLAT941_ALERT_3_G Average HKL Measurement Multiplicity ...........        1.9 Low
PLAT978_ALERT_2_G Number C-C Bonds with Positive Residual Density.          9 Info
PLAT992_ALERT_5_G Repd & Actual _reflns_number_gt Values Differ by          1 Check
```

```
   0 ALERT level A = Most likely a serious problem - resolve or explain
   0 ALERT level B = A potentially serious problem, consider carefully
   1 ALERT level C = Check. Ensure it is not caused by an omission or oversight
  11 ALERT level G = General information/check it is not something unexpected

   0 ALERT type 1 CIF construction/syntax error, inconsistent or missing data
   3 ALERT type 2 Indicator that the structure model may be wrong or deficient
   2 ALERT type 3 Indicator that the structure quality may be low
   5 ALERT type 4 Improvement, methodology, query or suggestion
   2 ALERT type 5 Informative message, check
```

It is advisable to attempt to resolve as many as possible of the alerts in all categories. Often the minor alerts point to easily fixed oversights, errors and omissions in your CIF or refinement strategy, so attention to these fine details can be worthwhile. In order to resolve some of the more serious problems it may be necessary to carry out additional measurements or structure refinements. However, the purpose of your study may justify the reported deviations and the more serious of these should normally be commented upon in the discussion or experimental section of a paper or in the "special_details" fields of the CIF. checkCIF was carefully designed to identify outliers and unusual parameters, but every test has its limitations and alerts that are not important in a particular case may appear. Conversely, the absence of alerts does not guarantee there are no aspects of the results needing attention. It is up to the individual to critically assess their own results and, if necessary, seek expert advice.

Publication of your CIF in IUCr journals

A basic structural check has been run on your CIF. These basic checks will be run on all CIFs submitted for publication in IUCr journals (*Acta Crystallographica*, *Journal of Applied Crystallography*, *Journal of Synchrotron Radiation*); however, if you intend to submit to *Acta Crystallographica Section C* or *E* or *IUCrData*, you should make sure that full publication checks are run on the final version of your CIF prior to submission.

Publication of your CIF in other journals

Please refer to the *Notes for Authors* of the relevant journal for any special instructions relating to CIF submission.

PLATON version of 16/07/2020; check.def file version of 12/07/2020

Datablock shi_4306_ - ellipsoid plot

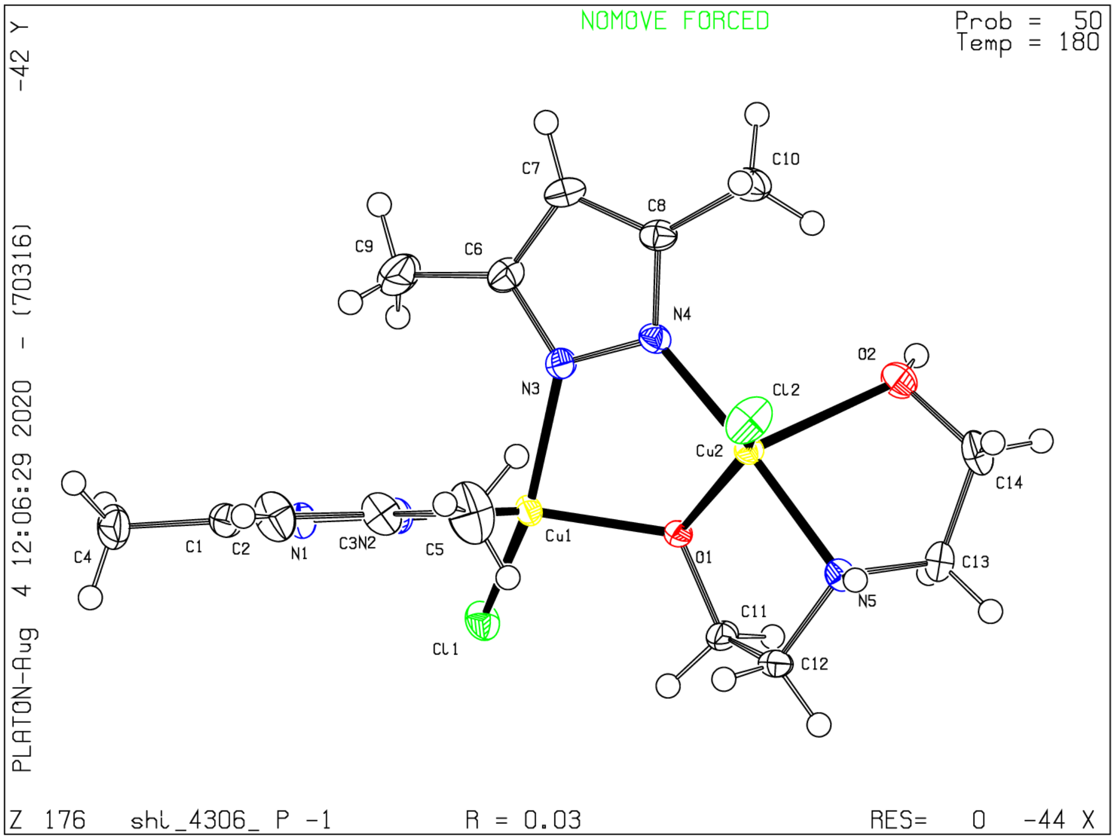

Supplement: Supplementary file 4 [file e-76-01503-sup3.pdf]
