# Supplementary material for: Crystal structure of di­chlorido-1κCl,2κCl-(μ2-3,5-dimethyl-1H-pyrazolato-1κN 2:2κN 1)(3,5-dimethyl-1H-pyrazole-2κN 2){μ-2-[(2-hy­droxy­eth­yl)amino-1κ2 N,O]ethano­lato-1:2κ2 O:O}dicopper(II)
Source: Acta Crystallogr E Crystallogr Commun. 2020 Aug 25;76(Pt 9):1503–7. doi: 10.1107/S2056989020011184 (PMC7472753; doi:10.1107/S2056989020011184)
Supplement: Supplementary file 6 [file e-76-01503-sup6.pdf]

Crystal structure of 2-Hydroxyethylamino-ethanolato-(3,5-dimethylpyrazole-N^2)-(μ_2-3,5-dimethylpyrazolato)-dichloride-di-copper(ii)

Oleksandr S. Vynohradov,[a] Vadim A. Pavlenko,[a] Inna S. Safyanova,[a] Sergiu Shova[b] and Safarmamad M. Safarmamadov[c]*

[a]Department of Chemistry, Taras Shevchenko National University of Kyiv, Volodymyrska str. 64/13, 01601 Kyiv, Ukraine, [b]"Poni Petru" Institute of Macromolecular Chemistry, Aleea Gr. Ghica, Voda 41A, 700487 Iasi, Romania, and [c]Department of Chemistry, Tajik National University, 17, Rudaki Avenue, Dushanbe, 734025, Tajikistan

Correspondence email: sash65@mail.ru;_linke33967@gmail.com

Abstract

The title compound, $Cu_2(C_5H_8N_2)(C_5H_7N_2)(C_4H_{10}NO_2)Cl_2$, is a pyrazolate aminoalcohol complex of the cyclic structure, which contains two dimethylpyrazole molecules in monodentate and bidentate-bridged coordination modes and monodeprotonated diethanolamine molecule. Both copper atoms are involved in the formation of nonplanar five-membered chelate rings. The Cu1 is in a distorted tetrahedral environment formed by the pyridine nitrogen atom of the protonated dimethylpyrazole molecule, the nitrogen atom of the deprotonated bridged dimethylpyrazole, the chlorine atom and the bridged oxygen atom of the monodeprotonated diethanolamine. The Cu2 has an intermediate environment between trigonal-bipyramidal and square-pyramidal, formed by the nitrogen atom of the deprotonated bridged dimethyl-pyrazole, the chlorine atom and the nitrogen atom of aminoalcohol, and two oxygen atoms of the deprotonated and protonated OH groups.

1. Chemical context

Metal complexes of paramagnetic metal ions formed by polynucleative or polydentate ligands are of great interest as they often exhibit nontrivial magnetic behaviour (Gumienna-Kontecka *et al.*, 2007; Suleimanov *et al.*, 2015; Gural'skiy *et al.*, 2012). Among polydentate and polynucleative ligands those containing both nitorgen and oxygen donor atoms are probably the most versatile and efficient chelators for the vast majority of metal ions (Pavlishchuk *et al.*, 2010; Strotmeyer *et al.*, 2003; Pavlishchuk *et al.*, 2011). Amino alcohol ligands and their derivatives are one of the most widely used representatives of N,O-chelators and attract attention as a strong polydentate ligands that can form coordination compounds with transition metals (Hughes *et al.*, 1972). Amino alcohols contain both amino and hydroxyl groups in one molecule, and therefore they are good chelating and bridging ligands. Polynuclear complexes of 3d metals with amino alcohols or their deprotonated forms can show non travial properties as catalysts, materials with different magnetic properties or biologically active compounds (Reiter *et al.*, 2006). Amino alcohol ligands are used to prepare copper (II) amino alcoholates, which, as a result of self-assembly, can form both mono- and multinuclear complexes. In bionuclear copper complexes, metal atoms can be connected by bridged oxygen atoms (alkoxy) from two different diethanolamine molecules (Tudor *et al.* 2003; Marin *et al.* 2005), or combined by a single oxygen atom from an amino alcohol and a bridged ligand molecule (Ashurov *et al.* 2015). There are several typical modes of binding of tridentate amino alcohol ligands to copper (II) ions and other metals such as lanthanides, yttrium, and alkaline earth metal (Breeze *et al.* 1994; Chen *et al.* 1995; Wang *et al.* 1995). It is a well-known fact that the copper coordination compounds can be modified with amino alcohols. For example, copper complexes with theophylline are promising objects due to their potential anti-tumor action and can be modified with diethanolamine by similar coordination of amino alcohols to the copper atom (Madarász *et al.*, 2000). Studies of both tridentate and bidentate-coordinated amino alcohol ligands to the copper atom

are being carried out (Wang 1995). Complexes of 3d-metals with a tricoordinated diethanolamine are interesting objects for synthesis and further studies (Buvaylo *et al.*, 2009). Considering the above, we understand the importance of accumulating a theoretical information base on such coordination compounds, and therefore in this article we report the synthesis and crystal structure of a new binuclear mixed-ligand copper (II) complex containing 3,5-dimethylpyrazole and diethanolamine.

2. Structural commentary

The crystal structure of title compound (Fig. 3) consists of dinuclear Cu_2(Hdmpz)(dmpz-H)(HDEA)Cl_2 (Hdmpz – 3,5-di-methyl-1H-pyrazole, dmpz-H – deprotonated 3,5-dimethyl-pyrazole, HDEA – monodeprotonated diethanolamine) units enclosed in two antisymmetrically oriented rows along the a axis. The unit cell consists of two unrelated structural fragments from both rows. Along the a axis within one row, each molecule is bonded to the preceding and subsequent hydrogen bonds of the same length. Along the b axis, the formation of molecules into dimers is due to the formation of hydrogen bonds of equal length between the bridged oxygen atom and the non deprotonated hydroxy group of the adjacent molecule. The title dinuclear pyrazolate aminoalcohol compound forms a cyclic structure. Two copper atoms with bridged oxygen atom of deprotonated diethanolamine and bridged molecule of deprotonated dimethylpyrazole form a five membered bimetallic cycle. Five-membered metallocycle has nonplanar structure. The nitrogen atoms of the bridged molecule of dimethylpyrazole are in the plane of the metallocycle while the bridged oxygen atom is out of this plane on 0.802 (1) Å. The angle between the Cu1O1Cu2 and Cu1Cu2N3N4 planes is 45.85 (8)°. Geometry environment of Cu1 with coordination number 4 is different from Cu2 with coordination number 5. The Cu1 is in a distorted tetra-hedral environment made by the pyridine nitrogen atom of the non deprotonated dimethylpyrazole molecule, the nitrogen atom of deprotonated bridged dimethylpyrazole, the chlorine atom and the bridged oxygen atom of monodeprotonated di-ethanolamine. The Cu2 has an intermediate environment between trigonal-bipyramidal and square pyramidal, formed by the nitrogen atom of deprotonated bridged dimethylpyrazole, the chlorine atom and the nitrogen atom of aminoalcohol, and two oxygen atoms of deprotonated and non deprotonated OH groups. The intermetallic distance between Cu1 and Cu2 is of 3.2439 (4) Å. The diethanolamine fragment is coordinated by all donor atoms to copper in a tetradentate mode and forms two similar non-planar five-membered metallocycles. It is to mentioned, that Cu2–O1 distance of 1.939 (1) Å differ significantly compared with Cu2–O2 of 2.244 (1) Å.

3. Supramolecular features

The hydrogen bonds (Table 2) are observed between the nitrogen and the chlorine atoms (N1–H···Cl2 and N5–H···Cl1) leading to the formation antisymmetric 1D-chains running along *a* axis. Two 1D-chains are connected together by hydrogen bonds between hydroxyl group as donor and O2 atom of the adjacent molecule as acceptor of proton.

4. Database survey

A search of the Cambridge Structural database (CSD version 5.41 (November 2019)) for the CuNH$(CCO)_2$ moiety (di-ethanolamine is coordinated to the copper atom) revealed one hundred and sixty-eight hits. Most similar to the title compound are the dinuclear complexes with coordinated two diethanolamine molecules, copper atoms are connected by a bridging oxygen atom and some another ligands (refcode ELESAP, Tudor *et al.*, 2003; refcode FARKAL, Marin *et al.*, 2005; refcode WITBAC, Madarasz *et al.*, 2000).

5. Synthesis and crystallization

1.76 mmol diethanolamine was added by drops to the 1.15 mmol acetonitrile solution of complex $Cu_4(\mu_2\text{-}Cl)_6(\mu_4\text{-}O)$ $(C_5H_8N_2)_4$ while stirring. The mixture stirred for 2 hours with oxygen access and without heating. Amino alcohol was

added to the brown solution and the color of mixture was changed to the green. Dark green crystals of $Cu_2(C_5H_8N_2)(C_5H_7N_2)(C_4H_{10}NO_2)Cl_2$ suitable for single crystal X-ray analysis were obtained by slow gas diffusion in an acetonitrile/hexane isolated system. Elemental analysis of $C_{14}H_{25}Cl_2Cu_2N_5O_2$: found C 33.96%, H 5.267% and N 14.13% (calculated C 34.08%, H 5.1%, N 14.19%). The yield was 55%. The starting $Cu_4(\mu_2\text{-}Cl)_6(\mu_4\text{-}O)(C_5H_8N_2)_4$ is a polymorphic modification of already known tetranuclear copper pyrazole-containing cluster $Cu_4OCl_6(C_5H_8N_2)_4$ and was obtained from Cu—$CuCl_2·2H_2O$—Hdmpz system.

6. Refinement

Crystal data, data collection and structure refinement details are summarized in Table 1.

Table 1

Experimental details

Crystal data	
Chemical formula	$C_{14}H_{25}Cl_2Cu_2N_5O_2$
M_r	493.37
Crystal system, space group	Triclinic, $P\overline{1}$
Temperature (K)	180
a, *b*, *c* (Å)	9.0732 (5), 10.7460 (6), 11.5578 (6)
α, β, γ (°)	92.373 (4), 102.383 (5), 112.703 (5)
V ($Å^3$)	1005.70 (10)
Z	2
Radiation type	Mo *K*α
μ (mm^{-1})	2.40
Crystal size (mm)	0.4 × 0.3 × 0.3
Data collection	
Diffractometer	Xcalibur, Eos
Absorption correction	Multi-scan<br>*CrysAlis PRO* 1.171.40.53 (Rigaku Oxford Diffraction, 2019) Empirical absorption correction using spherical harmonics, implemented in SCALE3 ABSPACK scaling algorithm.
T_{min}, T_{max}	0.553, 1.000
No. of measured, independent and observed [$I > 2\sigma(I)$] reflections	8833, 4681, 4108
R_{int}	0.018
$(\sin \theta/\lambda)_{max}$ ($Å^{-1}$)	0.693
Refinement	
$R[F^2 > 2\sigma(F^2)]$, $wR(F^2)$, S	0.027, 0.061, 1.05
No. of reflections	4681
No. of parameters	239
No. of restraints	3
H-atom treatment	H atoms treated by a mixture of independent and constrained refinement
$\Delta\rho_{max}$, $\Delta\rho_{min}$ (e $Å^{-3}$)	0.36, −0.43

Computer programs: *CrysAlis PRO* 1.171.40.53 (Rigaku OD, 2019), SHELXT (Sheldrick, 2015), *SHELXL* 2018/3 (Sheldrick, 2015), Olex2 1.3 (Dolomanov *et al.*, 2009).

Table 2

Hydrogen-bond geometry (Å, º) for (shi_4306_)

D—H···*A*	*D*—H	H···*A*	*D*···*A*	*D*—H···*A*
N1—H1···Cl2[i]	0.87 (2)	2.33 (2)	3.1201 (18)	152 (2)
N5—H5···Cl1[ii]	0.80 (2)	2.84 (2)	3.5593 (18)	150 (2)
O2—H2*A*···O1[iii]	0.85 (1)	1.88 (1)	2.7264 (19)	174 (2)

Symmetry codes: (i) $x+1, y, z$; (ii) $x-1, y, z$; (iii) $-x, -y, -z+1$.

References

Ashurov, J. M., Ibragimov, A. B. & Ibragimov, B. T. (2015). Polyhedron, 102, 441–446.

Breeze, S. R. & Wang, S. (1994). Inorg. chem., 33, 5113–5121.

Buvaylo, E. A., Kokozay, V. N., Vassilyeva, O. Y., Skelton, B. W. & Jezierska, J. (2009). Inorganica Chimica Acta, 362 (7), 2429–2434.

Chen, L., Breeze, S. R., Rousseau, R. J., Wang, S. & Thompson, L. K. (1995). Inorg. chem., 34, 454–465.

Dolomanov, O. V., Bourhis, L. J., Gildea, R. J., Howard, J. A. K. & Puschmann, H. (2009). *J. Appl. Cryst.* **42**, 339–341.

Gumienna-Kontecka, E., Golenya, I. A., Dudarenko, N. M., Dobosz, A., Haukka, M., Fritsky, I. O. & \'Swi\;atek-Koz\/lowska, J. (2007). *New J. Chem.*, 31, 1798-1805.

Gural'skiy, I. A., Quintero, C. M., Molnár, G., Fritsky, I. O., Salmon, L. & Bousseksou, A. (2012). *Chem. Eur. J.*, 18, 9946–9954.

Hughes, M. N., Waldron, B. & Rutt, K. J. (1972). Inorganica Chimica Acta, 6, 619–622.

Madarász, J., Bombicz, P., Czugler, M. & Pokol, G. (2000). Polyhedron, 19, 4, 457–463.

Marin, G., Tudor, V., Kravtsov, V. Ch., Schmidtmann, M., Simonov, Yu. A., Muller, A. & Andruh, M. (2005). Cryst. Growth. Des., 5(1), 279–282.

Pavlishchuk, A. V., Kolotilov, S. V., Zeller, M., Shvets, O. V., Fritsky, I. O., Lofland, S. E., Addison, A. W. & Hunter, A. D. (2011). Eur. J. Inorg. Chem., 31, 4826-4836.

Pavlishchuk, A. V., Kolotilov, S. V., Zeller, M., Thompson, L. K., Fritsky, I. O., Addison, A. W. & Hunter, A. D. (2010). *Eur. J. Inorg. Chem.*, 30, 4851-4858.

Reiter, L. G., Potaskalov, V. A., Andriiko, A. A., Kublanovsky, V. S., Pirskiy, Yu. K., Chmilenko, M. A., Lisin, V. I. & Chmilenko, S. M. (2006). Springer. Printed in the Netherlands, pp. 333–344.

Sheldrick, G. M. (2015). *Acta Cryst.* A**71**, 3–8.

Sheldrick, G. M. (2015). *Acta Cryst.* C**71**, 3–8.

Strotmeyer, K. P., Fritsky, I. O., Ott, R., Pritzkow, H. & Krämer, R. (2003). *Supramol. Chem*. **15**, 529-547.

Suleimanov, I., Kraieva, O., Sánchez Costa, J., Fritsky, I. O., Molnár, G., Salmon, L. & Bousseksou, A. (2015). *J. Mater. Chem. C*, 3, 5026–5032.

Tudor, V., Marin, G., Kravtsov, V., Simonov, Yu. A., Lipkowski, J., Brezeanu, M. & Andruh, M. (2003). Inorganica Chimica Acta, 353, 35–42.

Wang, S. (1995). J. Clust. Sci., 6(4), 463–484.

Wang, S., Pang, Z., Karen, D. L., Smith, K. D. L., Hua, Y.-S., Deslippe, C. & Wagner, M. J. (1995). Inorg. chem., 34, 908–917.

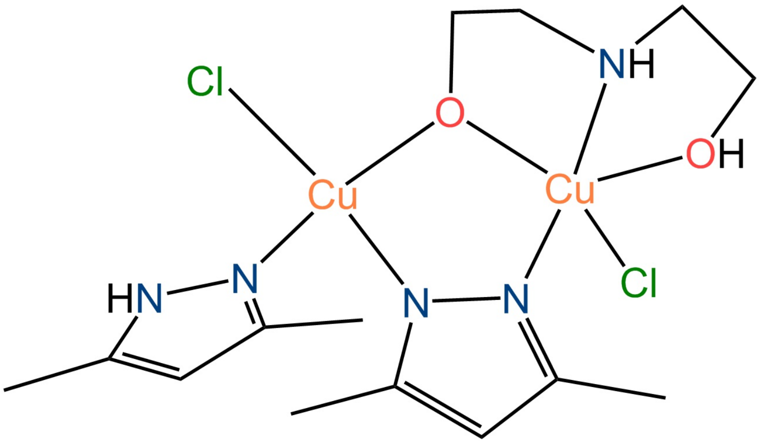

Figure 1

The structural formula of $Cu_2(C_5H_8N_2)(C_5H_7N_2)(C_4H_{10}NO_2)Cl_2$

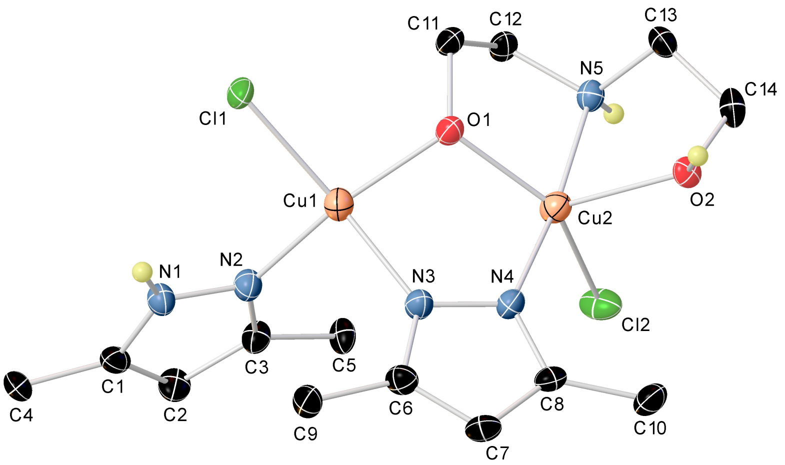

Figure 2

The molecular structure of $Cu_2(C_5H_8N_2)(C_5H_7N_2)(C_4H_{10}NO_2)Cl_2$

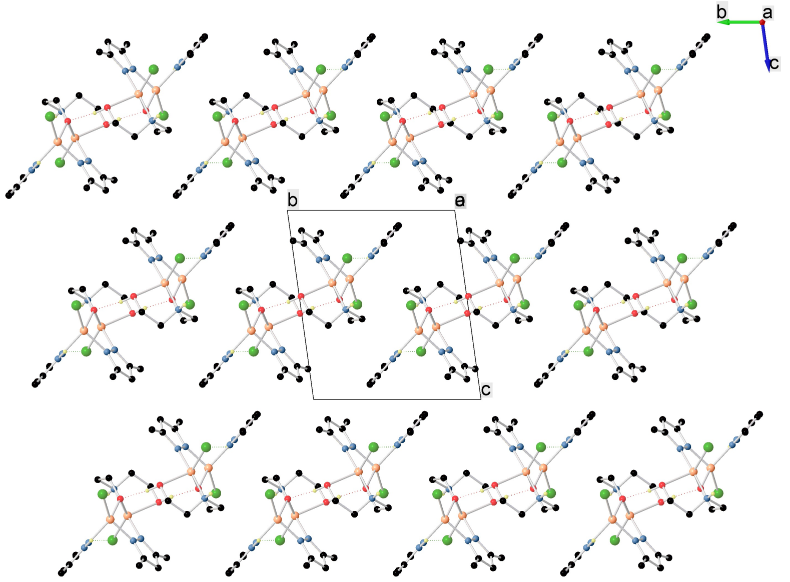

Figure 3

Crystal packing of the title compound viewed along the a axis direction

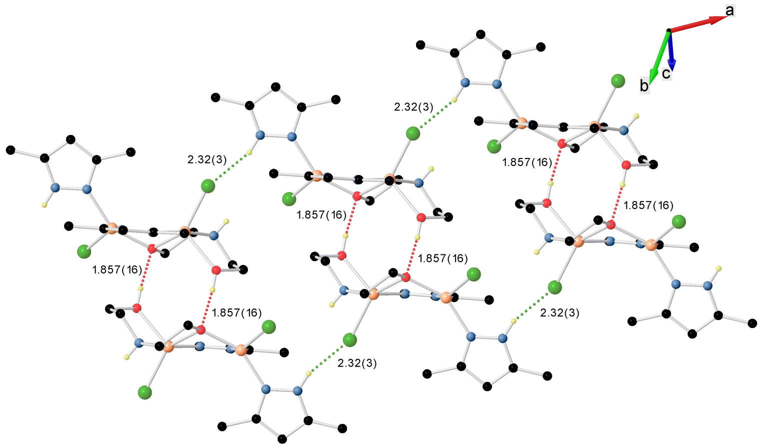

Figure 4

The illustration of hydrogen bonds in $Cu_2(C_5H_8N_2)(C_5H_7N_2)(C_4H_{10}NO_2)Cl_2$

supporting information

Crystal structure of 2-Hydroxyethylamino-ethanolato-(3,5-dimethylpyrazole-N^2)-(μ_2-3,5-dimethylpyrazolato)-dichloride-di-copper(ii)

Oleksandr S. Vynohradov, Vadim A. Pavlenko, Inna S. Safyanova, Sergiu Shova and Safarmamad M. Safarmamadov*

Computing details

Data collection: *CrysAlis PRO* 1.171.40.53 (Rigaku OD, 2019); cell refinement: *CrysAlis PRO* 1.171.40.53 (Rigaku OD, 2019); data reduction: *CrysAlis PRO* 1.171.40.53 (Rigaku OD, 2019); program(s) used to solve structure: SHELXT (Sheldrick, 2015); program(s) used to refine structure: *SHELXL* 2018/3 (Sheldrick, 2015); molecular graphics: Olex2 1.3 (Dolomanov *et al.*, 2009); software used to prepare material for publication: Olex2 1.3 (Dolomanov *et al.*, 2009).

(shi_4306_)

Crystal data

$C_{14}H_{25}Cl_2Cu_2N_5O_2$
M_r = 493.37
Triclinic, $P\bar{1}$
a = 9.0732 (5) Å
b = 10.7460 (6) Å
c = 11.5578 (6) Å
α = 92.373 (4)°
β = 102.383 (5)°
γ = 112.703 (5)°
V = 1005.70 (10) Å^3
Z = 2
F(000) = 504
D_x = 1.629 Mg m^{-3}
Mo $K\alpha$ radiation, λ = 0.71073 Å
Cell parameters from 4259 reflections
θ = 1.8–29.2°
μ = 2.40 mm^{-1}
T = 180 K
Prism, clear intense green
0.4 × 0.3 × 0.3 mm

Data collection

Xcalibur, Eos diffractometer
Radiation source: fine-focus sealed X-ray tube, Enhance (Mo) X-ray Source
Graphite monochromator
Detector resolution: 16.1593 pixels mm^{-1}
ω scans
Absorption correction: multi-scan *CrysAlis PRO* 1.171.40.53 (Rigaku Oxford Diffraction, 2019) Empirical absorption correction using spherical harmonics, implemented in SCALE3 ABSPACK scaling algorithm.
T_{min} = 0.553, T_{max} = 1.000
8833 measured reflections
4681 independent reflections
4108 reflections with $I > 2\sigma(I)$
R_{int} = 0.018
θ_{max} = 29.5°, θ_{min} = 1.8°
h = −11→10
k = −13→13
l = −15→15

Refinement

Refinement on F^2
Least-squares matrix: full
$R[F^2 > 2\sigma(F^2)]$ = 0.027
$wR(F^2)$ = 0.061
S = 1.05
4681 reflections
239 parameters
3 restraints
Primary atom site location: dual
Hydrogen site location: mixed

H atoms treated by a mixture of independent and constrained refinement
$w = 1/[\sigma^2(F_o^2) + (0.0223P)^2 + 0.5005P]$ where $P = (F_o^2 + 2F_c^2)/3$
$(\Delta/\sigma)_{max} = 0.001$
$\Delta\rho_{max} = 0.36$ e Å^{-3}
$\Delta\rho_{min} = -0.43$ e Å^{-3}

Special details

Geometry. All esds (except the esd in the dihedral angle between two l.s. planes) are estimated using the full covariance matrix. The cell esds are taken into account individually in the estimation of esds in distances, angles and torsion angles; correlations between esds in cell parameters are only used when they are defined by crystal symmetry. An approximate (isotropic) treatment of cell esds is used for estimating esds involving l.s. planes.

Fractional atomic coordinates and isotropic or equivalent isotropic displacement parameters (Å^2) for (shi_4306_)

	x	*y*	*z*	U_{iso}*/U_{eq}
C1	0.8325 (3)	0.5970 (2)	0.87558 (18)	0.0213 (4)
C2	0.7173 (3)	0.6360 (2)	0.9068 (2)	0.0303 (5)
H2	0.737259	0.705862	0.966030	0.036*
C3	0.5648 (3)	0.5504 (2)	0.8325 (2)	0.0249 (5)
C4	1.0153 (3)	0.6516 (2)	0.9206 (2)	0.0299 (5)
H4A	1.053223	0.583541	0.900423	0.045*
H4B	1.045118	0.675213	1.005925	0.045*
H4C	1.065349	0.731079	0.884536	0.045*
C5	0.3987 (3)	0.5503 (3)	0.8250 (3)	0.0427 (7)
H5A	0.360893	0.577420	0.750146	0.064*
H5B	0.405616	0.612959	0.889610	0.064*
H5C	0.322664	0.460389	0.830295	0.064*
C6	0.3858 (3)	0.1471 (2)	0.84323 (18)	0.0222 (5)
C7	0.2607 (3)	0.0678 (2)	0.89308 (19)	0.0237 (5)
H7	0.271158	0.021651	0.958574	0.028*
C8	0.1163 (3)	0.0714 (2)	0.82511 (17)	0.0189 (4)
C9	0.5663 (3)	0.1797 (3)	0.8802 (2)	0.0411 (7)
H9A	0.604540	0.168147	0.810855	0.062*
H9B	0.585441	0.119540	0.934917	0.062*
H9C	0.624799	0.272188	0.918419	0.062*
C10	−0.0560 (3)	0.0091 (2)	0.8396 (2)	0.0265 (5)
H10A	−0.073035	0.071365	0.891799	0.040*
H10B	−0.072923	−0.073948	0.873356	0.040*
H10C	−0.132710	−0.010057	0.762932	0.040*
C11	0.1643 (3)	0.2883 (2)	0.41712 (17)	0.0192 (4)
H11A	0.113125	0.213856	0.351586	0.023*
H11B	0.265007	0.353315	0.401922	0.023*
C12	0.0478 (3)	0.3565 (2)	0.42571 (19)	0.0209 (4)
H12A	0.107248	0.443231	0.477042	0.025*
H12B	0.002000	0.373194	0.347020	0.025*
C13	−0.2144 (2)	0.1487 (2)	0.39115 (18)	0.0210 (4)
H13A	−0.163870	0.108472	0.343581	0.025*
H13B	−0.289446	0.177492	0.337517	0.025*
C14	−0.3067 (3)	0.0466 (2)	0.4624 (2)	0.0234 (5)
H14A	−0.363395	0.085052	0.505581	0.028*
H14B	−0.388495	−0.033863	0.409321	0.028*
Cl1	0.57083 (6)	0.33924 (5)	0.50253 (4)	0.02235 (12)
Cl2	−0.05593 (7)	0.31818 (6)	0.74647 (5)	0.02968 (14)

Cu1	0.41923 (3)	0.31469 (2)	0.63633 (2)	0.01473 (7)
Cu2	0.02665 (3)	0.20789 (2)	0.61602 (2)	0.01395 (7)
N1	0.7493 (2)	0.49360 (18)	0.78604 (15)	0.0175 (4)
H1	0.788 (3)	0.446 (2)	0.749 (2)	0.021*
N2	0.5853 (2)	0.46264 (17)	0.75898 (15)	0.0182 (4)
N3	0.3203 (2)	0.19520 (17)	0.74924 (14)	0.0172 (4)
N4	0.1545 (2)	0.14748 (17)	0.73807 (14)	0.0166 (3)
N5	−0.0860 (2)	0.26688 (17)	0.47523 (15)	0.0156 (3)
H5	−0.133 (3)	0.308 (2)	0.499 (2)	0.019*
O1	0.20146 (16)	0.23756 (13)	0.52738 (11)	0.0144 (3)
O2	−0.19285 (18)	0.01036 (14)	0.54515 (13)	0.0228 (3)
H2A	−0.200 (2)	−0.0673 (12)	0.5176 (19)	0.034*

Atomic displacement parameters (Å²) for (shi_4306_)

	U^{11}	U^{22}	U^{33}	U^{12}	U^{13}	U^{23}
C1	0.0227 (11)	0.0187 (11)	0.0162 (10)	0.0030 (8)	0.0029 (8)	0.0002 (8)
C2	0.0311 (13)	0.0258 (12)	0.0283 (12)	0.0063 (10)	0.0088 (10)	−0.0104 (10)
C3	0.0250 (12)	0.0215 (11)	0.0293 (12)	0.0088 (9)	0.0111 (9)	−0.0017 (9)
C4	0.0221 (12)	0.0329 (13)	0.0233 (12)	0.0027 (10)	−0.0004 (9)	−0.0024 (10)
C5	0.0312 (15)	0.0431 (16)	0.0576 (18)	0.0179 (12)	0.0162 (13)	−0.0080 (14)
C6	0.0232 (11)	0.0251 (12)	0.0208 (11)	0.0128 (9)	0.0044 (8)	0.0053 (9)
C7	0.0303 (12)	0.0264 (12)	0.0174 (10)	0.0135 (10)	0.0071 (9)	0.0093 (9)
C8	0.0247 (11)	0.0188 (10)	0.0149 (10)	0.0090 (9)	0.0076 (8)	0.0027 (8)
C9	0.0271 (14)	0.0578 (19)	0.0427 (15)	0.0214 (13)	0.0059 (11)	0.0249 (13)
C10	0.0276 (12)	0.0299 (12)	0.0251 (11)	0.0108 (10)	0.0132 (9)	0.0106 (9)
C11	0.0163 (10)	0.0270 (11)	0.0155 (10)	0.0085 (9)	0.0058 (8)	0.0080 (8)
C12	0.0192 (11)	0.0214 (11)	0.0231 (11)	0.0078 (8)	0.0067 (8)	0.0115 (9)
C13	0.0162 (10)	0.0233 (11)	0.0206 (10)	0.0076 (8)	0.0003 (8)	−0.0015 (9)
C14	0.0148 (10)	0.0187 (11)	0.0344 (12)	0.0053 (8)	0.0054 (9)	−0.0027 (9)
Cl1	0.0171 (3)	0.0277 (3)	0.0228 (3)	0.0076 (2)	0.00912 (19)	0.0017 (2)
Cl2	0.0361 (3)	0.0433 (3)	0.0217 (3)	0.0284 (3)	0.0089 (2)	−0.0001 (2)
Cu1	0.01202 (13)	0.01529 (13)	0.01474 (12)	0.00332 (9)	0.00345 (9)	0.00108 (9)
Cu2	0.01386 (13)	0.01565 (13)	0.01402 (12)	0.00676 (10)	0.00508 (9)	0.00341 (9)
N1	0.0144 (9)	0.0178 (9)	0.0193 (9)	0.0061 (7)	0.0034 (7)	−0.0003 (7)
N2	0.0153 (9)	0.0193 (9)	0.0192 (9)	0.0059 (7)	0.0052 (7)	0.0006 (7)
N3	0.0149 (9)	0.0198 (9)	0.0177 (8)	0.0073 (7)	0.0047 (7)	0.0048 (7)
N4	0.0160 (9)	0.0173 (9)	0.0175 (8)	0.0063 (7)	0.0069 (7)	0.0041 (7)
N5	0.0144 (9)	0.0148 (9)	0.0192 (9)	0.0068 (7)	0.0060 (7)	0.0014 (7)
O1	0.0129 (7)	0.0162 (7)	0.0143 (7)	0.0053 (5)	0.0046 (5)	0.0047 (5)
O2	0.0245 (8)	0.0128 (7)	0.0268 (8)	0.0043 (6)	0.0040 (6)	0.0010 (6)

Geometric parameters (Å, º) for (shi_4306_)

C1—C2	1.372 (3)	C11—H11B	0.9700
C1—C4	1.490 (3)	C11—C12	1.517 (3)
C1—N1	1.341 (3)	C11—O1	1.432 (2)
C2—H2	0.9300	C12—H12A	0.9700
C2—C3	1.393 (3)	C12—H12B	0.9700
C3—C5	1.490 (3)	C12—N5	1.473 (3)
C3—N2	1.335 (3)	C13—H13A	0.9700

C4—H4A	0.9600	C13—H13B	0.9700
C4—H4B	0.9600	C13—C14	1.499 (3)
C4—H4C	0.9600	C13—N5	1.478 (2)
C5—H5A	0.9600	C14—H14A	0.9700
C5—H5B	0.9600	C14—H14B	0.9700
C5—H5C	0.9600	C14—O2	1.432 (3)
C6—C7	1.386 (3)	Cl1—Cu1	2.2403 (6)
C6—C9	1.494 (3)	Cl2—Cu2	2.2937 (6)
C6—N3	1.344 (3)	Cu1—N2	1.9635 (16)
C7—H7	0.9300	Cu1—N3	1.9770 (17)
C7—C8	1.391 (3)	Cu1—O1	1.9388 (13)
C8—C10	1.494 (3)	Cu2—N4	1.9268 (17)
C8—N4	1.341 (3)	Cu2—N5	1.9916 (17)
C9—H9A	0.9600	Cu2—O1	2.0001 (13)
C9—H9B	0.9600	Cu2—O2	2.2441 (14)
C9—H9C	0.9600	N1—H1	0.87 (2)
C10—H10A	0.9600	N1—N2	1.353 (2)
C10—H10B	0.9600	N3—N4	1.363 (2)
C10—H10C	0.9600	N5—H5	0.80 (2)
C11—H11A	0.9700	O2—H2A	0.853 (9)

C2—C1—C4	131.7 (2)	N5—C12—H12B	109.9
N1—C1—C2	106.26 (19)	H13A—C13—H13B	108.4
N1—C1—C4	122.0 (2)	C14—C13—H13A	110.0
C1—C2—H2	126.7	C14—C13—H13B	110.0
C1—C2—C3	106.60 (19)	N5—C13—H13A	110.0
C3—C2—H2	126.7	N5—C13—H13B	110.0
C2—C3—C5	129.4 (2)	N5—C13—C14	108.28 (16)
N2—C3—C2	109.4 (2)	C13—C14—H14A	109.8
N2—C3—C5	121.1 (2)	C13—C14—H14B	109.8
C1—C4—H4A	109.5	H14A—C14—H14B	108.3
C1—C4—H4B	109.5	O2—C14—C13	109.25 (16)
C1—C4—H4C	109.5	O2—C14—H14A	109.8
H4A—C4—H4B	109.5	O2—C14—H14B	109.8
H4A—C4—H4C	109.5	N2—Cu1—Cl1	96.92 (5)
H4B—C4—H4C	109.5	N2—Cu1—N3	96.00 (7)
C3—C5—H5A	109.5	N3—Cu1—Cl1	144.30 (5)
C3—C5—H5B	109.5	O1—Cu1—Cl1	98.89 (4)
C3—C5—H5C	109.5	O1—Cu1—N2	148.51 (7)
H5A—C5—H5B	109.5	O1—Cu1—N3	86.76 (6)
H5A—C5—H5C	109.5	N4—Cu2—Cl2	95.29 (5)
H5B—C5—H5C	109.5	N4—Cu2—N5	171.92 (7)
C7—C6—C9	129.3 (2)	N4—Cu2—O1	87.45 (6)
N3—C6—C7	108.86 (19)	N4—Cu2—O2	99.70 (6)
N3—C6—C9	121.9 (2)	N5—Cu2—Cl2	92.21 (5)
C6—C7—H7	127.0	N5—Cu2—O1	84.77 (6)
C6—C7—C8	105.95 (19)	N5—Cu2—O2	81.29 (6)
C8—C7—H7	127.0	O1—Cu2—Cl2	142.41 (4)
C7—C8—C10	130.0 (2)	O1—Cu2—O2	112.12 (5)
N4—C8—C7	108.08 (19)	O2—Cu2—Cl2	104.35 (4)
N4—C8—C10	121.88 (19)	C1—N1—H1	127.8 (15)

C6—C9—H9A	109.5	C1—N1—N2	111.74 (17)
C6—C9—H9B	109.5	N2—N1—H1	120.4 (15)
C6—C9—H9C	109.5	C3—N2—Cu1	129.29 (15)
H9A—C9—H9B	109.5	C3—N2—N1	105.97 (16)
H9A—C9—H9C	109.5	N1—N2—Cu1	124.74 (13)
H9B—C9—H9C	109.5	C6—N3—Cu1	132.39 (15)
C8—C10—H10A	109.5	C6—N3—N4	107.94 (17)
C8—C10—H10B	109.5	N4—N3—Cu1	119.66 (13)
C8—C10—H10C	109.5	C8—N4—Cu2	132.78 (14)
H10A—C10—H10B	109.5	C8—N4—N3	109.16 (16)
H10A—C10—H10C	109.5	N3—N4—Cu2	117.88 (13)
H10B—C10—H10C	109.5	C12—N5—C13	115.41 (16)
H11A—C11—H11B	108.3	C12—N5—Cu2	105.06 (12)
C12—C11—H11A	109.9	C12—N5—H5	111.3 (17)
C12—C11—H11B	109.9	C13—N5—Cu2	111.35 (13)
O1—C11—H11A	109.9	C13—N5—H5	106.2 (16)
O1—C11—H11B	109.9	Cu2—N5—H5	107.3 (16)
O1—C11—C12	108.86 (16)	C11—O1—Cu1	122.25 (12)
C11—C12—H12A	109.9	C11—O1—Cu2	111.54 (11)
C11—C12—H12B	109.9	Cu1—O1—Cu2	110.88 (6)
H12A—C12—H12B	108.3	C14—O2—Cu2	104.53 (12)
N5—C12—C11	108.97 (17)	C14—O2—H2A	108.4 (13)
N5—C12—H12A	109.9	Cu2—O2—H2A	131.5 (13)

C1—C2—C3—C5	−177.8 (3)	C9—C6—C7—C8	179.7 (2)
C1—C2—C3—N2	0.1 (3)	C9—C6—N3—Cu1	−0.9 (3)
C1—N1—N2—C3	0.9 (2)	C9—C6—N3—N4	179.7 (2)
C1—N1—N2—Cu1	−178.30 (15)	C10—C8—N4—Cu2	2.8 (3)
C2—C1—N1—N2	−0.8 (3)	C10—C8—N4—N3	177.58 (18)
C2—C3—N2—Cu1	178.58 (16)	C11—C12—N5—C13	−76.2 (2)
C2—C3—N2—N1	−0.5 (3)	C11—C12—N5—Cu2	46.87 (17)
C4—C1—C2—C3	178.4 (2)	C12—C11—O1—Cu1	−111.63 (16)
C4—C1—N1—N2	−179.0 (2)	C12—C11—O1—Cu2	22.96 (19)
C5—C3—N2—Cu1	−3.4 (3)	C13—C14—O2—Cu2	41.26 (17)
C5—C3—N2—N1	177.5 (2)	C14—C13—N5—C12	161.80 (17)
C6—C7—C8—C10	−177.5 (2)	C14—C13—N5—Cu2	42.16 (19)
C6—C7—C8—N4	1.0 (2)	Cu1—N3—N4—C8	−178.77 (13)
C6—N3—N4—C8	0.7 (2)	Cu1—N3—N4—Cu2	−3.11 (18)
C6—N3—N4—Cu2	176.41 (13)	N1—C1—C2—C3	0.5 (3)
C7—C6—N3—Cu1	179.33 (14)	N3—C6—C7—C8	−0.6 (2)
C7—C6—N3—N4	−0.1 (2)	N5—C13—C14—O2	−56.9 (2)
C7—C8—N4—Cu2	−175.87 (14)	O1—C11—C12—N5	−46.9 (2)
C7—C8—N4—N3	−1.1 (2)		

Hydrogen-bond geometry (Å, º) for (shi_4306_)

D—H···*A*	*D*—H	H···*A*	*D*···*A*	*D*—H···*A*
N1—H1···Cl2^{i}	0.87 (2)	2.33 (2)	3.1201 (18)	152 (2)
N5—H5···Cl1^{ii}	0.80 (2)	2.84 (2)	3.5593 (18)	150 (2)
O2—H2*A*···O1^{iii}	0.85 (1)	1.88 (1)	2.7264 (19)	174 (2)

Symmetry codes: (i) $x+1, y, z$; (ii) $x-1, y, z$; (iii) $-x, -y, -z+1$.
